# Distraction driven by reward history: Attentional capture and sequential effects

**DOI:** 10.3758/s13414-025-03167-7

**Published:** 2026-02-23

**Authors:** Andrea De Cesarei, Serena Mastria, Maurizio Codispoti

**Affiliations:** Department of Psychology, University of Bologna, Viale Berti Pichat, 5, Bologna, BO Italy

**Keywords:** Attentional capture, Reward learning, Binding-retrieval, Intertrial priming, Attentional suppression, Distractor repetition

## Abstract

Values learned through previous experiences of reward can later modulate attentional capture if associated with a distractor in singleton search tasks (value-driven attentional capture; VDAC). Moreover, it has been shown that re-encountering distractor features can facilitate performance or reduce attentional capture (sequential effects). However, little is known about how sequential effects and attentional capture are jointly modulated by learned distractor value. Here, we examined the role of learned reward in sequential modulation of attentional capture. In two experiments we used a VDAC paradigm, varying the type of reward (monetary vs. sustainability-related). After associating letter colors with a high or low reward, or none at all, in a flanker task (learning phase), in a subsequent singleton task (test phase) we manipulated the effects of distractor value of the present and of the previous trial on attentional capture. In both experiments repetition of the same distractor value from trial N-1 to trial N was associated with faster responses, and reward value did not modulate this facilitation. In addition, attentional capture by rewarded, compared with unrewarded, distractors was observed when the preceding trial was unrewarded. Value-signaling distractors, if re-encountered, reduced attentional capture in the current trial, and this happened even for rewarded distractors of different values (e.g., high value followed by low value, and vice versa). These results suggest that, for different forms of incentives, repetition of previously rewarded distractors and attentional capture by the current reward interact in modulating the processing of learned values.

## Introduction

Every day we are exposed to a massive amount of visual information from the environment, some of which is relevant for our survival, while the remaining input is irrelevant and may lead to distraction. Given that our processing resources are limited, the human attentional system selects and inhibits information based on top-down goals and bottom-up factors such as physical salience, novelty, or emotional significance, resulting in an adaptive guidance of behavior but also in undesired attentional capture when distracting information is presented (Codispoti et al., 2016; Corbetta & Shulman, 2002; Serences & Yantis, 2007; Theeuwes, 2010).

In addition to the attentional guidance and capture in the ongoing trial, it has been shown that features from the current trial can modulate future encounters with the same stimulus. Some examples of such sequential effects are intertrial priming effects, where processing of stimulus features is facilitated upon their repetition (Becker et al., 2009; Kristjánsson et al., 2007; Lamy et al., 2006; Leber & Egeth, 2006; Maljkovic & Nakayama, 1994). In the context of the study of cognitive control and of action control, a comprehensive model has been suggested (Frings et al., 2020) that aims to include perceptual, attentional, and response components of sequential effects. Based on previous theoretical suggestions (Hommel, 2004; Kahneman et al., 1992), this model states that stimulus features and responses are integrated into memory entries or “event files” that are automatically retrieved when individuals encounter stimuli that match any features of previous episodes. For instance, in tasks requiring responses to experimental stimuli, if a trial feature is repeated (as opposed to changed) in two neighboring trials, the retrieval of the elements of the previous episode can facilitate the current performance (Hommel, 1998, 2002, 2004; Frings et al., 2020; see also Pastötter & Frings, 2018). Interestingly, both intertrial priming and feature-binding effects have been shown for both target and distractor stimuli (Belopolsky et al., 2008; Eimer et al., 2010; Feldmann-Wüstefeld & Schubö, 2016; Frings et al., 2007, 2020; Kristjánsson & Driver, 2008; Lamy et al., 2008; Maljkovic & Nakayama, 1994). In attentional capture tasks, the representation of previous distractors is thought to be adaptive, in that it might allow for a more efficient rejection of these distractors in future encounters. Moreover, some studies have observed that the process of encoding information in memory (Gong & Li, 2014; Infanti et al., 2015; see also Awh et al., 2006; Gazzaley & Nobre, 2012; Zanto et al., 2011) can be modulated by the rewarding value of events. However, it is not clear what the role of stimulus relevance (e.g., reward-relatedness) is in the modulation of sequential effects, and of binding-retrieval mechanisms, for distractors in attentional capture tasks.

While some stimuli or events capture attention because of their bottom-up salience (e.g., abrupt onset) or of top-down factors (e.g., expectations), other stimuli are salient because of their history of selections or history of rewards, or because they signal the magnitude of a to-be-delivered reward (Awh et al., 2012; Failing & Theeuwes, 2017, 2018). Specifically, the learned association of a stimulus with a rewarding experience (for reviews, see Anderson, 2015; Bourgeois et al., 2016; Failing & Theeuwes, 2018) can modulate visual selective attention, and this effect is long lasting (Chelazzi et al., 2013; Della Libera & Chelazzi, 2009; Engelmann & Pessoa, 2007; Raymond & O’Brien, 2009). The deployment of attention, and the way it is modulated by reward as well as by previous selections, plays a crucial role in the interaction with the environment, and has been linked to sign-tracking in the animal learning literature (Boakes, 1977), and to the mechanisms involved in drug addiction in humans (Flagel et al., 2009; Tomie et al., 2008). Paradigms that investigate value-driven attentional capture (VDAC; Anderson, 2013) modulate the rewarding value of experimental stimuli either through a two-phase procedure (learning and test; e.g., Anderson et al., 2011), or through Pavlovian conditioning in a single phase, i.e., with relevant stimuli signaling not the delivery but the magnitude of rewards (e.g., Le Pelley et al., 2015). Even when no more rewards are delivered (two-phase procedures) or despite value-signaling stimuli being task irrelevant (single-phase procedures), relevant stimuli still retain the ability to capture attention (Anderson, 2016; Anderson & Halpern, 2017; Anderson & Yantis, 2013; Anderson et al., 2011; Chelazzi et al., 2013; Failing & Theeuwes, 2017; Jiao et al., 2015; Le Pelley et al., 2015). These results suggest that value-signaling stimuli are preferentially processed in the visual environment and capture attention in a non-voluntary way even when these contrast with the current task goal.

Here, we investigated the role of learned value in the sequential modulation of attentional capture. To this end, we asked participants to take part in a learning phase in which stimuli could be associated with a high reward, a low reward, or no reward. In the subsequent test session, we manipulated the value of the distractor both in the ongoing trial (attentional capture by distractor reward) and in the preceding trial (hence resulting in distractor condition repetition or change depending on the match between the rewarding value of the previous and current distractor). To manipulate reward history, we replicated the paradigm proposed by Mine and Saiki (2015, 2018). In this paradigm participants perform a flanker task in the reward-learning phase, in which the letter color, which was irrelevant for target selection, predicted either a monetary reward or no reward. In the test phase, we manipulated the rewarding value of both the current and the previous trial in a singleton task (Theeuwes, 1991), and focused on the role of value in the sequential modulation of (value-driven) attentional capture. On the one hand (aspecific scenario), the repetition (vs. change) of any distractor feature (i.e., color) across successive trials might speed up the current performance (i.e., priming effects; e.g., Kristjánsson & Driver, 2008; Lamy et al., 2008; Pastötter & Frings, 2018), irrespective of learned value. On the other hand (reward-specific scenario), one might expect that value-signaling distractors may capture attention to different extents based on the value and the repetition/change of the preceding distractor (e.g., Gong & Li, 2014; Hickey et al., 2010, 2014; Infanti et al., 2015).

## Experiment 1

### Method

#### Participants

A total of 69 participants took part in Experiment 1 (41 women, 28 men; M_age_ = 23.24, SD_age_ = 3.49 years). The sample size was determined based on an a priori power analysis (MorePower 6.0.4; Campbell & Thompson, 2012). To detect the reported effect of partial eta squared =.06 (medium effect size; Cohen, 1988) with 80% power (α =.05) in a 3 × 3 (Current Distractor Value × Preceding Distractor Value) repeated-measures analysis of variance (RM-ANOVA), the analysis indicated a minimum of 48 participants, consistent with previous studies that examined value-driven attentional capture (e.g., Anderson, 2016; Anderson et al., 2011; Mine & Saiki, 2015; Sha & Jiang, 2016). All participants had self-reported normal or corrected-to-normal visual acuity and color vision, and none of them reported current or past neurological or psychopathological problems. The participants had no previous experience with the materials used in this experiment. Informed consent was obtained from all participants. This study was not preregistered. The experimental protocol was in accordance with the tenets of the Declaration of Helsinki and was approved by the local Ethics Committee.

#### Apparatus

A computer equipped with Open Sesame 3.11 (Mathôt et al., 2012) was used to present the stimuli. Participants viewed the monitor from a distance of 57 cm. Average room illumination was ~ 80 lx, as measured by a diode-type digital luxmeter. Head position was maintained using an adjustable chin rest. Manual responses were recorded using a standard keyboard (Z and M keys).

#### General procedure

**Fig. 1:**
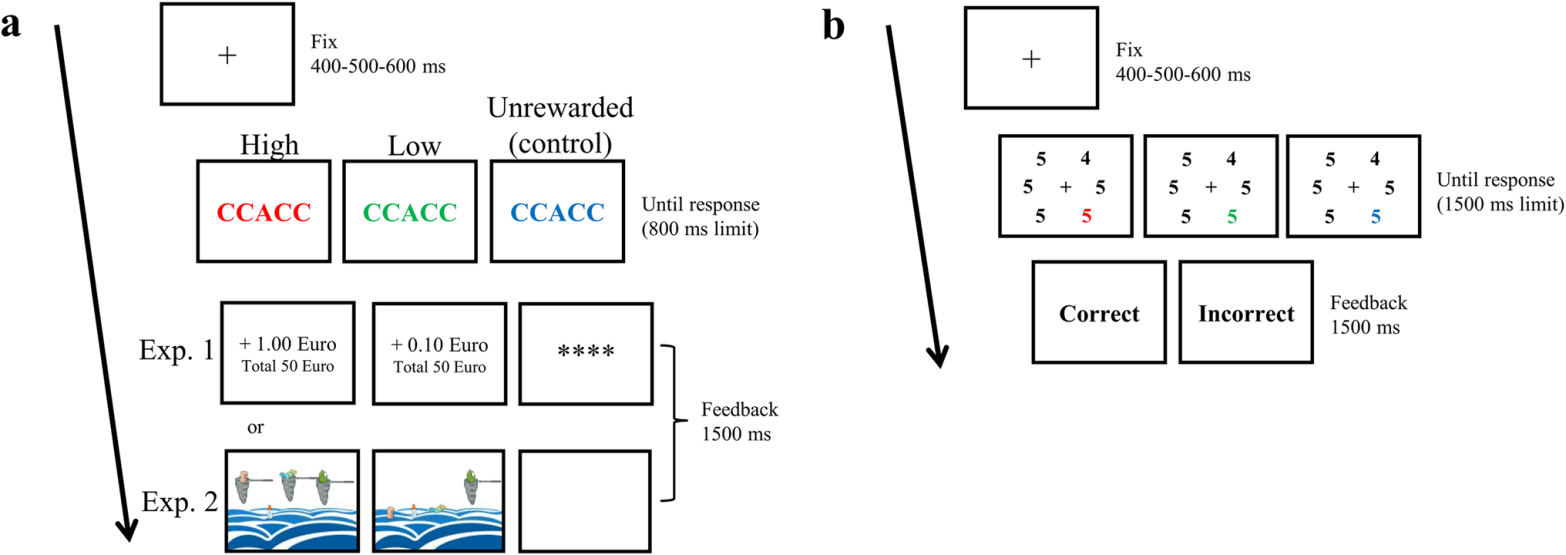
Trial structure of the value-driven attentional capture (VDAC) paradigm. (**a**) In the learning phase (flanker task), participants made a two-alternative forced-choice response on the central letter (target) identity according to the response mapping explained at the beginning of each block. Only the feedback screens differed between experiments: The feedback display in Experiment 1 indicated the monetary gain for the trial and the cumulative total amount earned, while the feedback display in Experiment 2 indicated the point gain (drawn as bins of plastic collected from the sea) for each accurate trial, which then translated into a donation to an association fighting pollution. (**b**) In the test phase (singleton search task), participants were asked whether the different number (target) in the search display was an odd or an even number. One of the five remaining numbers (distractors) was colored (red, green, or blue), and only corrective feedback was provided

The procedure is shown in Fig. 1. First, in the rewarded learning phase participants performed a flanker task (Eriksen & Eriksen, 1974). Then, in the unrewarded test phase (see Mine & Saiki, 2015), participants performed an additional singleton visual search task (Theeuwes, 1991, 1992). The session took about 50 min.

#### Learning phase

##### Stimuli

Each trial consisted of a fixation display, a flanker display, and a feedback display. The fixation display contained a white fixation cross (0.5° × 0.5° visual angle) displayed in the center of a black screen. This was followed by a flanker display that contained a target letter in the center of the display (visual angle; 1.1° horizontal × 1.4° vertical), flanked to the left and right by other letters of equal size (1.4° center-to-center). All letters (both targets and flankers) were of the same color, which could be either red, green, or blue. We used 16 uppercase letters of the alphabet (excluding AIJLMNOQWZ), divided into four groups of four letters. When participants made a correct response, the feedback display indicated the earnings for the trial. When the response was incorrect, or no response was made, the feedback displayed “Incorrect.”

##### Design and procedure

The training phase consisted of 240 trials, divided into four experimental blocks of 60 trials each (a total of 80 trials for each condition: high reward, low reward, and unrewarded control). Each block was preceded by ten practice trials. Participants took a short break after each block. In each experimental block, a different four-letter group was selected to avoid a perceptual learning effect for any specific group of letters (Mine & Saiki, 2015), and two letters were assigned to each response key, “Z” or “M”. For instance, in a block including V, R, C, and T, if the central target was V or R participants had to respond using the “Z” key, and if the target was C or T they had to respond using the “M” key. In congruent trials, the response mapping for the target and distracting flankers was compatible (e.g., RRVRR), whereas in incongruent trials, the response mapping for the target and distracting flankers was incompatible (e.g., CCVCC). Notably, the target and distracting flankers were always different letters (e.g., RRRRR was never presented). Participants made a two-alternative response on target identity (the central letter) according to the response mapping that was explained at the beginning of each block. The block order and response mapping were counterbalanced across participants. As depicted in Fig. 1a, each trial began with the presentation of the fixation display for a randomly varying interval of 400, 500, or 600 ms. The flanker display was shown until participants made a response or the trial timed out (800 ms), followed by a black screen for 1,000 ms. Thereafter, visual feedback was presented for 1,500 ms.

In the high-reward condition, high-reward feedback was given in 75% of the trials and low-reward feedback in the remaining 25% of the trials; for the low-reward condition, the probability of reward association (high and low) was reversed. In the control condition, four asterisks were presented as feedback for all the correct trials (i.e., “****”). The assignment of red, green, and blue to high-value, low-value, and unrewarded control colors was fully counterbalanced across participants. Participants were told that color was irrelevant to the task and instructed to respond as quickly as possible while minimizing errors. Although the probability of reward association (high and low) was the same (high reward in 75% of the trials and low reward in 25% of the trials), the feedback display indicating the earnings for the trial slightly varied across participants. For 13 participants, the high- and low-reward feedback consisted of an earning of €0.03 and €0.01, respectively. For 21 participants the high- and low-reward feedback consisted of a symbolic earning of €0.03 and €0.01 (i.e., no real money compensation was delivered), of which they were made aware at the beginning of the experimental session. For 35 participants, the high- and low-reward feedback consisted again of a symbolic earning of €1.00 and €0.10, respectively (see Fig. 1a; the feedback pertaining to Experiment 1). This difference was aimed at understanding the most effective reward manipulation in modulating VDAC based on: (1) the actual delivery of money (tangible vs. virtual), as it has been shown that VDAC can also be observed with non-tangible rewards, such as points (Sha & Jiang, 2016) or social rewards (Anderson, 2016), and (2) the relative value of the monetary reward (i.e., high vs. low), as it has been shown that rewards must exceed a certain minimal threshold to produce consistent effects on the development of attentional bias (Anderson & Halpern, 2017). While the monetary and symbolic rewards were conceptually similar, prior to the main analysis we ran an ANOVA with the Earning Group factor (three levels: €0.03 vs. €0.01 with monetary compensation; €0.03 vs. €0.01 without monetary compensation; €1.00 vs. €0.10 without monetary compensation) as a between-subjects factor. Results showed no significant interactions as a function of Earning condition in either the learning, *F*s < 2.34, *p*s > 0.058, η^2^_p_s > 0.06, or the test, *F*s < 1.79, *p*s > 0.13, η^2^_p_s = 0.05, phases.

#### Test phase

##### Stimuli

Each trial consisted of a fixation display, a search array, and a feedback display. The fixation display contained a white fixation cross (0.5° × 0.5° visual angle) in the center of a black screen. Then, a search display consisting of a fixation cross surrounded by six numbers (1.3° horizontal × 1.6° vertical visual angle) placed at equal intervals around an imaginary circle with a 6° radius was presented. The six numbers in the display included one target and five non-targets. All non-targets represented the same digit, while the target was a different digit. The target and four of the five non-targets were white, whereas the remaining non-target (from now on, “distractor”) was red, green, or blue. The numbers used for the target and non-targets ranged from 2 to 9. One number was assigned to each target and non-target (e.g., 2 for the target and 9 for non-targets), so that in half of the trials the numbers were all odd or all even, and in the other half of the trials the target was odd and the non-targets were even, or vice versa. Target parity (odd or even), target location, distractor color, and distractor location were counterbalanced across participants. Visual similarity between targets and non-targets was prevented by excluding the following combinations: 6 and 3, 6 and 5, 8 and 9, 8 and 3, and 3 and 9.

##### Design and procedure

The test phase consisted of 192 trials, divided into four experimental blocks of 48 trials each (a total of 64 trials for each condition: high, low, and unrewarded control); this test phase was preceded by a learning phase consisting of ten practice trials. Participants took a short break after each block. As shown in Fig. 1b, each trial began with the presentation of the fixation display for a randomly varying interval of 400, 500, or 600 ms. The search array then appeared and remained on-screen until a response was made or 1,500 ms had elapsed. Participants were asked whether the target was an odd (“Z” key) or even (“M” key) number. The block order and response mapping were counterbalanced across participants. Unlike the learning phase, no reward feedback was given during the test phase, but visual feedback informing participants whether their response on each trial was correct or incorrect was presented. Participants were told that color was irrelevant to the task and that they should respond as quickly as possible while minimizing errors.

#### Data analysis

Practice trials, the first trial of each block, errors, and trials following an error, were excluded from the analysis of both the learning and test phase. For each participant response times (RTs) of more than 2.5 absolute deviations above or below the median (MADs) of their respective condition (Current Distractor Value: high, low, unrewarded control) were excluded. We employed the MAD procedure because it is a more robust measure with which to detect outliers than that of excluding values over two or three standard deviations around the mean, and is immune to sample size (Leys et al., 2013). In the training phase, the average number of discarded trials per participant was 56 (out of 240). In the test phase, the average number of discarded trials per participant was 50 (out of 192).

For all analyses, repeated-measures analyses of variance (ANOVAs) were carried out on RTs, with Current Distractor Value (high, low, unrewarded control) and Preceding Distractor Value (high, low, unrewarded control) as within-subjects factors, using Huynh–Feldt correction when appropriate. The partial eta squared statistic (η^2^_p_), indicating the proportion between the variance explained by one experimental factor and the total variance, was calculated and reported. If a superordinate main effect or interaction was significant, we proceeded with ANOVAs on subordinate conditions or carried out post hoc comparisons.

### Results

#### Learning

The averaged accuracy across all participants was high in both the learning and the test phase for the two experiments (Table 1). We conducted a two-way ANOVA on RTs with Reward (high, low, unrewarded control) and Congruency (congruent, incongruent) as within-subjects factors in the training phase. There were significant main effects of Congruency, *F*(1, 68) = 152.34, *p* <.001, *η*^2^_p_ =.69, indicating faster responses for congruent (M = 515.00 ms, SD = 53.40 ms) compared with incongruent (M = 536.61 ms, SD = 53.98 ms, *p* <.001) trials, and Reward, *F*(2, 136) = 6.28, *p* =.002, *η*^2^_p_ =.08, but no interaction, *F*(2, 136) =.33, *p* =.714, *η*^2^_p_ <.01 (Table 2). Post hoc comparisons regarding Reward revealed significantly faster RTs for both high- (M = 523.62 ms, SD = 52.07 ms) and low-reward (M = 523.79 ms, SD = 54.98 ms) conditions compared with the unrewarded control condition (M = 529.99 ms, SD = 55.23 ms, ps <.003), with no difference between high- and low-reward conditions (p =.937).

**Table 1:** Mean accuracy (%) and standard deviation (in brackets) in the training and test phases

	Training	Test
Experiment 1	88.90 (6.72)	88.22 (6.26)
Experiment 2	90.41 (4.70)	89.71 (6.11)

**Table 2:** Mean response times (in ms) and standard deviation (in brackets) in the training phase

	High reward		Low reward		Unrewarded Control	
	Congruent	Incongruent	Congruent	Incongruent	Congruent	Incongruent
Experiment 1	512.16 (52.91)	535.08 (53.88)	512.92 (53.75)	534.66 (57.92)	519.90 (56.93)	540.07 (55.89)
Experiment 2	458.54 (61.73)	484.06 (63.90)	457.34 (60.84)	485.11 (64.05)	456.67 (63.55)	484.29 (65.12)

#### Test

**Fig. 2:**
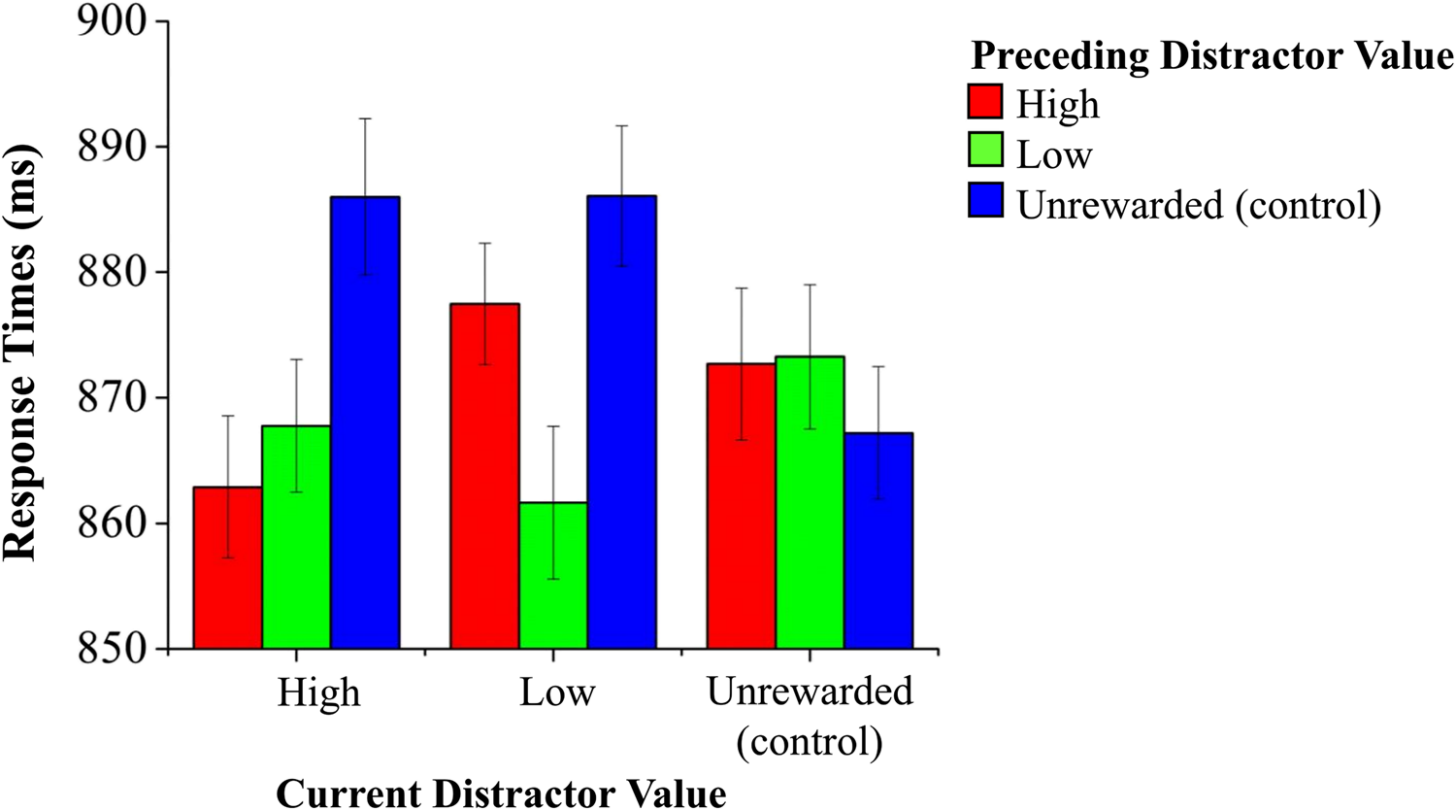
Response times in Experiment 1. Error bars represent within-participants standard error of the mean (O’Brien & Cousineau, 2014)

Results of the test phase are reported in Fig. 2. In the test phase, no main effect of Current Distractor Value, *F*(2, 136) =.28, *p* =.752, *η*^2^_p_ <.01, emerged. However, a significant main effect of Preceding Distractor Value, *F*(2, 136) = 3.39, *p* =.037, *η*^2^_p_ =.04, and interaction between Current Distractor Value and Preceding Distractor Value, *F*(4, 272) = 4.03, *p* =.003, *η*^2^_p_ =.05, emerged.

In terms of the analysis of each level of current distractor value, when the present trial was high-value, *F*(2, 136) = 5.36, *p* =.008, *η*^2^_p_ =.07, participants were faster if the preceding trial was either high- or low-value, than when it was unrewarded, *F*s(1, 68) > 5.29, *p*s <.024, *η*^2^_p_s >.07; the former two conditions did not differ significantly from each other, *F*(1, 68) =.70, *p* =.406, *η*^2^_p_ =.01. When the ongoing trial was low-value, a significant effect of Preceding Distractor Value, *F*(2, 136) = 5.59, *p* =.005, *η*^2^_p_ =.07, indicated that participants were faster at reporting the target when the preceding trial was low-value than when it was either high-value or an unrewarded control, *F*s(1, 68) > 4.57, *p*s <.036, *η*^2^_p_s >.06; the latter two conditions did not significantly differ from each other, *F*(1, 68) = 1.24, *p* =.269, *η*^2^_p_ =.01. Finally, no significant effect of Preceding Distractor Value, *F*(2, 136) =.40, *p* =.666, *η*^2^_p_ <.01, emerged when the current distractor was an unrewarded color.

Analyzing each previous distractor condition separately, a significant main effect of Current Distractor Value was observed in trials preceded by an unrewarded distractor color, *F*(2, 136) = 3.88, *p* =.023, *η*^2^_p_ =.05, indicating that participants were slower at reporting the target in both high-and low-value trials compared with unrewarded trials, *F*s(1, 68) > 5.86, *p*s <.018, *η*^2^_p_s >.07; the former two conditions did not significantly differ from each other, *F*(1, 68) <.01, *p* =.993, *η*^2^_p_ <.01. In contrast, no significant effect of Current Distractor Value was observed for trials preceded by either high-value or low-value distractors, *F*s(2, 136) < 1.94, *p*s >.148, *η*^2^_p_s <.02.

As conditions that were repeated from trial N-1 to trial N appeared to be faster compared with change conditions, a further analysis was carried out in terms of change or repetition from the previous to the current trial, with a two-way ANOVA with factors Preceding Distractor Value (three levels: high value, low value, unrewarded control) and Change (Repeat/Change) as within-subjects factors. A highly significant effect of Change was observed, *F*(1, 68) = 15.31, *p* <.001, *η*^2^_p_ =.18, indicating faster responses when color was repeated (M = 863.92, SD = 79.72) compared with when changed (M = 877.85, SD = 79.617), and neither significant effects of Preceding Distractor Value, *F*(2,136) = 2.16, *p* =.119, *η*^2^_p_ =.03, nor a significant interaction, *F*(2,136) =.54, *p* =.581, *η*^2^_p_ <.01.

### Discussion

In Experiment 1, we investigated sequential modulation of attentional capture in a task in which the learned reward value of distractors was manipulated. Focusing on the intertrial effects of repetition (change vs. repetition), consistent with previous studies on intertrial priming, change trials were associated with slower RTs compared with trials in which distractor value repeated (Belopolsky et al., 2008; Eimer et al., 2010; Feldmann-Wüstefeld & Schubö, 2016; Frings et al., 2007, 2020; Kristjánsson & Driver, 2008; Lamy et al., 2006, 2008; Maljkovic & Nakayama, 1994). Here, change/repetition concerned the change in distractor from trial N-1 to trial N, consistent with previous studies that observed intertrial effects with distractor stimuli. Interestingly, when investigating the effects of current distractor as a function of the preceding one, we observed similar RTs for current unrewarded distractors regardless of the preceding distractor condition. Furthermore, when the current distractor was high-value, distractor repetition effect generalized to preceding distractors that were either high- or low-value.

The rewarding value of the current distractor modulated attentional capture, with slower responses for high and low reward compared with control distractors when the previous trial was an unrewarded distractor. In contrast, no attentional capture pattern was observed for trials preceded by either high-value or low-value distractors. This pattern of results is consistent with the idea that upon repetition, retrieval of a previous relevant (interference- or conflict- related) feature may reactivate a protective setting that prevents attentional capture from emerging (Pastötter & Frings, 2018; see also Frings et al., 2020; Hommel, 1998), and extends previous results on distractor-repetition effects (Feldmann-Wüstefeld & Schubö, 2016; Kristjánsson & Driver, 2008; Lamy et al., 2008) by incorporating the rewarding value of distractors into the factors that modulate sequential effects (Gong & Li, 2014; Infanti et al., 2015).

In Experiment 2, we replicated the design of Experiment 1, with the exception that a different reward was provided. Previous studies observed VDAC mainly with monetary rewards, but also with other types of symbolic reward such as social rewards (Anderson, 2015). The degree to which a contextually irrelevant stimulus comes to acquire value via reward learning, and subsequently modulates attention, may indeed change as a function of the perception of the value itself. In Experiment 2 we employed an incentive that has a symbolic meaning which varies considerably between individuals, i.e., environmental sustainability (i.e., the gratification derived from undertaking environmental sustainability practices). We therefore presented the reward as sustainability-relevant points and examined whether the individual sensitivity to environmental issues might impact attentional capture. On the one hand (reward-general scenario), it is possible that the numerical consistence of the reward rather than the subjective importance of environmental issues modulates the sequential modulation of attentional capture mechanisms. On the other hand (sustainability-specific scenario), since sensitivity to one such value varies significantly from one person to the other, individual sensitivity to environmental sustainability may modulate the sequential modulation of attentional capture, and “green reward” effects may be evident only in participants who recognize the importance of environmental protection.

## Experiment 2

### Method

#### Participants

A total of 110 participants took part in Experiment 2 (72 women, 38 men; M_age_ = 24.33, SD = 5.86 years). We were interested in testing potential individual differences in reward sensitivity and observing the reported effect of partial eta squared =.06 (medium effect size; Cohen, 1988) with.80 probability (α =.05) in a 3 × 3 × 3 (Sustainability Attitude Level × Current Distractor Value × Preceding Distractor Value) repeated-measures analysis of variance (IM-RM-ANOVA). According to MorePower 6.0.4 (Campbell & Thompson, 2012), a sample size of 66 participants is recommended. The actual sample size was comparable or superior to that of previous studies that examined value-driven attentional capture (e.g., Anderson, 2016; Anderson et al., 2011; Mine & Saiki, 2015; Sha & Jiang, 2016). All participants reported normal or corrected-to-normal visual acuity and color vision, and none of them reported current or past neurological or psychopathological problems. The participants had no previous experience with the materials used in this experiment. Informed consent was obtained from all participants. This study was not preregistered. The experimental protocol conforms to the Declaration of Helsinki and was approved by the local Ethics Committee.

#### Apparatus

In Experiment 2, the apparatus was identical to that of Experiment 1.

#### General procedure

The procedure was similar to the previous experiment, except that a different type of reward feedback, i.e., environmental sustainability, was used in the learning phase (see Mine & Saiki, 2015). Before starting with this, participants were introduced to a rapid stream of 90 pictures depicting sea plastic pollution (each picture appeared for 666 ms; total time 1 min). Participants were told that a monetary donation to an association fighting plastic pollution would be made based on their performance in the task. They were explicitly informed that for each correct response a score would be assigned, and that a donation to the association would be established based on the total gained score. Participants were also required to fill out two questionnaires measuring their attitudes toward environmental issues, i.e., the Pro-Environmental Behaviours (PEBs) Scale (Menardo et al., 2020) and the Attitudes toward Sustainable Development (ASD) Scale (Biasutti & Frate, 2017). The entire session took about 1.5 h.

#### Learning phase

##### Stimuli, design, and procedure

In Experiment 2, the stimuli, design, and procedure were similar to those of Experiment 1. Notably, as depicted in Fig. 1a (see the feedback pertaining to Experiment 2), when participants made a correct response, the feedback display consisted of a stylized image depicting either: (a) three nets collecting a large quantity of plastic from the sea, meaning that 3 points had been earned, which then translated into a larger donation to the association (high-reward feedback); or (b) one net collecting a small quantity of plastic from the sea, meaning that 1 point had been earned, which then translated into a smaller donation to the association (low-reward feedback); or (c) a blank screen meaning the trial was not counted, and therefore did not result in a sum being donated to the association (unrewarded control condition). When the response was incorrect, or no response was made, the visual feedback displayed the sea polluted by plastic (without any nets) meaning that a donation, that could have been earned had a correct response been given, was not achieved. In the high-reward condition, high-reward feedback (i.e., the image depicting three nets collecting a large quantity of plastic from the sea) was given in 75% of the trials and low-reward feedback (i.e., the image depicting one net collecting a small amount of plastic from the sea) in the remaining 25% of the trials; for the low-reward condition, the probability of reward association (high and low) was reversed. In the control condition, a blank screen was presented as feedback for all the correct trials, meaning these trials did not result in a sum being donated to the association. Assignment of red, green, and blue to high-value, low-value, and unrewarded control colors was fully counterbalanced across participants. Participants were instructed that color was irrelevant to the task and that they should respond as quickly as possible while minimizing errors.

#### Test phase

##### Stimuli, design, and procedure

The test phase was the same as in Experiment 1 (see Fig. 1b).

#### Data analysis

Similar to Experiment 1, practice trials, the first trial of each block, errors, and trials following an error, were excluded from the analysis of both the learning and the test phase. For each participant RTs of over 2.5 MADs above or below the median of their respective condition (Current Distractor Value: high, low, unrewarded control) were excluded. In the learning phase, the average number of discarded trials per participant was 52 (out of 240). In the test phase, the average number of discarded trials per participant was 46 (out of 192).

Concerning statistics, the same analytical designs as in Experiment 1 were adopted. Moreover, to assess whether value-driven attentional capture could change as a function of individuals’ attitudes toward environmental issues, both the PEBS and ASD scores were first divided into tertiles (low, medium, and high), and then submitted to a 3 (Sustainability Attitude Level) × 3 (Current Distractor Value) × 3 (Preceding Distractor Value) ANOVA on RTs in the test phase, with the first variable as a between-subjects factor and the latter as within-subjects factors. No significant main effects of the between-participant factor (*F*s <.52, *p*s >.592, *η*^2^_p_s <.01) or interactions involving sustainability attitudes (*F*s <.76, *p*s >.635, *η*^2^_p_s <.01) were observed. Therefore, all the analyses described below do not include the Sustainability Attitude Level as a between-subjects factor.

### Results

#### Learning

In the training phase, the ANOVA on RTs with Reward (high, low, unrewarded control) and Congruency (congruent, incongruent) as within-subjects factors revealed a significant main effect of Congruency only, *F*(1, 109) = 451.33, *p* <.001, *η*^2^_p_ =.80, indicating faster responses for congruent (M = 457.52 ms, SD = 60.83 ms) compared with incongruent (M = 484.49 ms, SD = 62.82 ms, p < 0.001) trials, with no significant main effect of Reward, *F*(2, 218) =.13, *p* =.873, *η*^2^_p_ <.01, or interaction, *F*(2, 218) =.54, *p* =.583, *η*^2^_p_ <.01.

#### Test

**Fig. 3:**
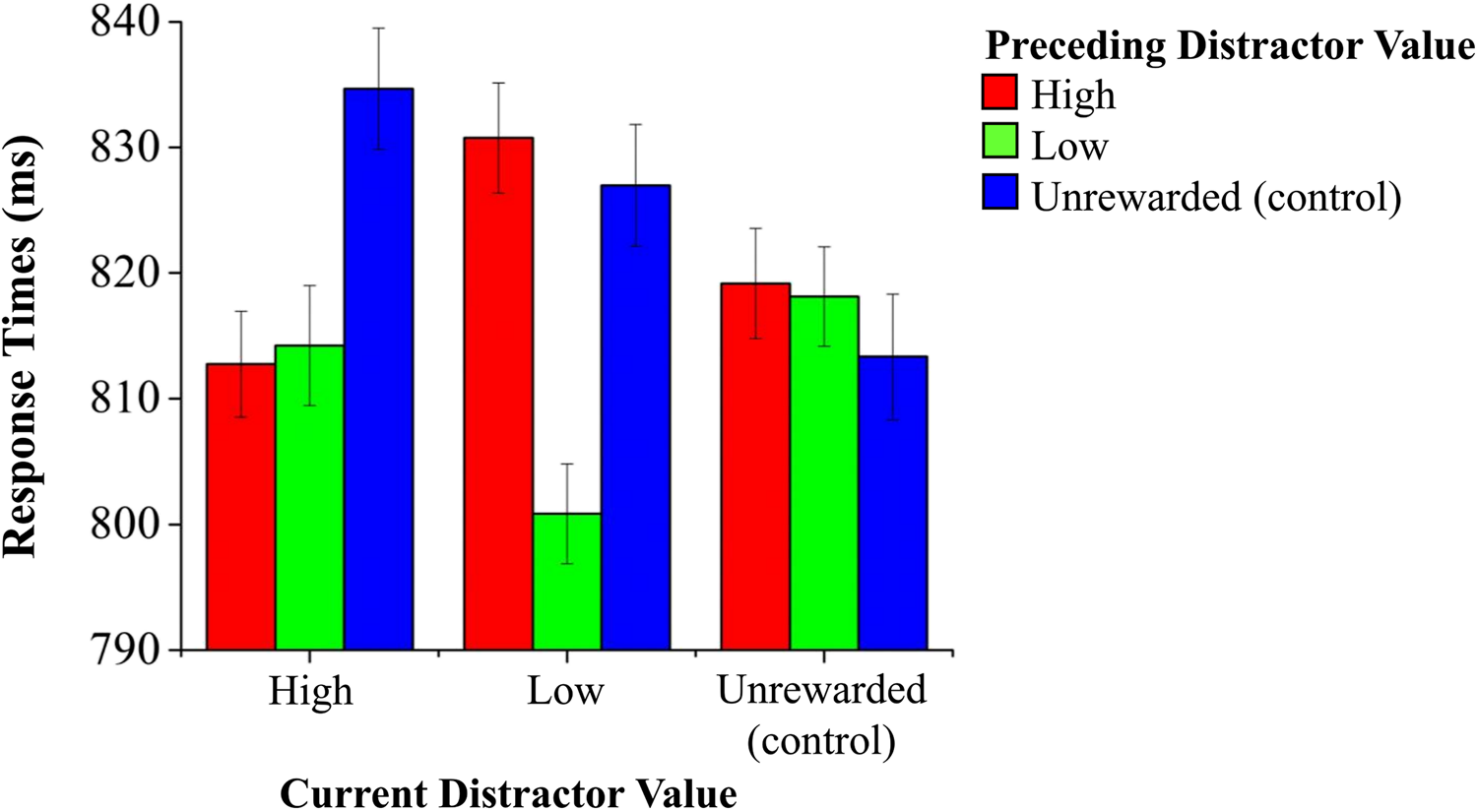
Response times in Experiment 2. Error bars represent within-participants standard error of the mean (O’Brien & Cousineau, 2014)

Results of the test phase are given in Fig. 3. No significant main effect of Current Distractor Value, *F*(2, 218) =.42, *p* =.651, *η*^2^_p_ <.01, was observed. However, a significant main effect of Preceding Distractor Value, *F*(2, 218) = 6.66, *p* =.002, *η*^2^_p_ =.05, and interaction between Current and Preceding Distractor Value, *F*(4, 436) = 8.25, *p* <.001, *η*^2^_p_ =.07, were observed. The pattern of results of Experiment 2 was similar to that of Experiment 1. Based on this significant interaction and analyzing each current distractor value level separately, when the ongoing trial was high-value, a significant effect of Preceding Distractor Value was observed, *F*(2, 218) = 7.56, *p* =.001, *η*^2^_p_ =.06, revealing that participants were faster when the preceding trial was either high- or low-value than when it was unrewarded, *F*s(1, 109) > 8.88, *p*s <.004, *η*^2^_p_s >.07; the former two conditions did not significantly differ from each other, *F*(1, 109) =.07, *p* =.792, *η*^2^_p_ <.01. When the ongoing trial was low-value, a significant effect of Preceding Distractor Value, *F*(2, 218) = 14.10, *p* <.001, *η*^2^_p_ =.11, indicated that participants were faster at reporting the target when the preceding trial was low-value, rather than either high-value, *F*(1, 109) = 24.14, *p* <.001, *η*^2^_p_ =.18, or unrewarded, *F*(1, 109) = 19.63, *p* <.001, *η*^2^_p_ =.15; the latter two conditions did not significantly differ from each other, *F*(1, 109) =.34, *p* =.556, *η*^2^_p_ <.01. Finally, no effect of preceding distractor condition was observed when the current distractor condition was an unrewarded control, *F*(1, 218) =.56, *p* =.567, *η*^2^_p_ <.01.

As in Experiment 1, we also examined the significant interaction based on the value of the preceding trial. Again, in trials following distractors of the unrewarded color a significant main effect of Current Distractor Value was observed, *F*(2, 218) = 5.11, *p* =.007, *η*^2^_p_ =.04, indicating slower RTs in both high- and low- value, compared with unrewarded, trials, *F*s(1, 109) > 4.45, *p*s <.037, *η*^2^_p_s >.03; the former two conditions did not significantly differ from each other, *F*(1, 109) = 1.17, *p* =.281, *η*^2^_p_ =.01. When preceding distractor value was either high-value or low-value, significant effects of Current Distractor Value were observed, *F*s(2, 218) > 4.40, *p*s <.013, *η*^2^_p_s >.03. In trials preceded by high-value distractors, current high-value distractors were associated with faster responses than low-value distractors, *F*(1, 109) = 9.46, *p* =.003, *η*^2^_p_ =.08, with no other significant differences, *F*s(1, 109) < 3.19, *p*s >.077, *η*^2^_p_s <.02. In trials preceded by low-value distractors, both current high-value and unrewarded distractors were slower than current low-value distractors, *F*s(1, 109) > 5.35, *p*s <.023, *η*^2^_p_s >.04, with no difference between current high-value and unrewarded distractors, *F*(1, 109) =.39, *p* =.529, *η*^2^_p_ <.01.

As conditions that were repeated from trial N-1 to trial N appeared to be faster compared with change conditions, a further analysis was carried out in terms of change or repetition from the previous to the current trial, with a two-way ANOVA with Preceding Distractor Value (three levels: high-value, low-value, unrewarded control) and Change (Repeat/Change) as within-subjects factors. A highly significant effect of Change was observed, *F*(1, 109) = 49.90, *p* <.001, *η*^2^_p_ =.31, indicating faster responses when color was repeated (M = 808.98, SD = 102.98) compared with when changed (M = 825.59, SD = 101.42), and a significant effect of Preceding Distractor Value, *F*(2, 218) = 5.90, *p* =.004, *η*^2^_p_ =.05, with faster RTs for low distractor value compared with both high-value and unrewarded distractors, *F*s(1, 109) > 8.72, *p*s <.004, *η*^2^_p_s >.07, and no difference between high-value and unrewarded distractors, *F*(1, 109) =.43, *p* =.510, *η*^2^_p_ <.01. No significant interaction, *F*(2, 218) =.18, *p* =.819, *η*^2^_p_ <.01, emerged.

### Discussion

Experiment 2 replicated Experiment 1, using environmental sustainability rewards as feedback during the learning phase. Here, we were interested in understanding whether such a reward, the value of which is not recognized by everyone, is able to exert general or individual-specific effects on the sequential modulation of attentional capture.

A reward-general scenario was observed, and no effects of individuals’ attitudes toward environmental issues were found on the sequential modulation of attentional capture. The pattern of results of Experiment 2 replicated Experiment 1, suggesting that the sequential modulation of attentional capture can be effectively modulated by different kinds of rewards, including monetary and sustainability-related rewards.

#### Combined analysis: Feature-change and attentional capture

The results of Experiments 1 and 2 are consistent in revealing an interaction of Preceding and Current Distractor Value, which results from a highly significant effect of distractor change/repetition, as well as from a distinct attentional capture that is unique to when the preceding distractor value is unrewarded. To further establish these two effects, we carried out two additional analyses on the combined data from Experiments 1 and 2; in these analyses, Experiment was kept as an additional between-participant factor, that was never observed to interact with any of the other considered factors.

**Fig. 4:**
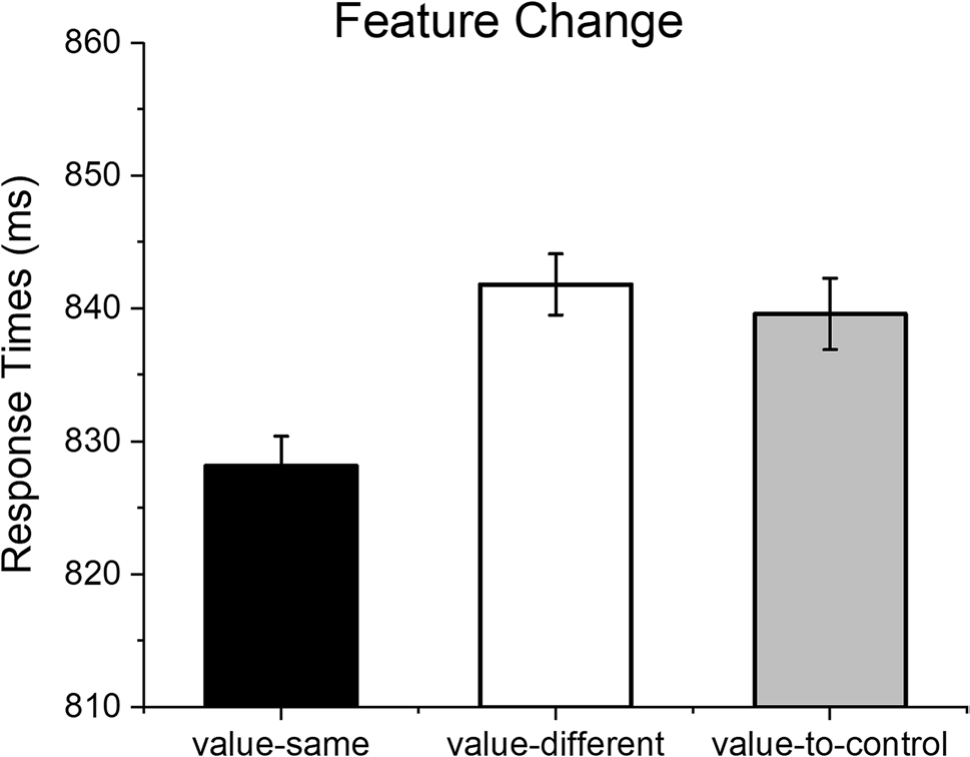
The effects of feature change (from a valued preceding distractor to different distractor conditions in the actual trial). The distractor condition (high-value, low-value, unrewarded) in the N-1 trial in this analysis is the same across the three conditions (value-same, value-different, value-to-control)

In the first analysis (feature change; Fig. 4), the aim was to investigate the effects of change from a valued distractor (e.g., a high-value one) to a distractor of the same value (value-same), a different value (value-different), or an unrewarded distractor (value-to-control). Therefore, in this analysis the value of the distractor in the preceding trial was kept constant, while the value of the distractor in the current trial was varied. The reasoning was that, if the effects of value change/repetition are specific to distractor condition, then similarly slower responses for value-different and value-to-control, compared with value-same, conditions should be observed, as in both cases distractors change from a valued one (either high or low) to a different distractor condition. On the other hand, if value contributes to repetition effects, then one can expect a more graded result, with fastest responses to value-same condition, intermediate to value-different, and slowest to value-to-control conditions, consistent with the change in value from trial N-1 to trial N. A main effect of Distractor Change was observed, *F*(2, 354) = 10.93, *p* <.001, *η*^2^_p_ =.05, indicating slower responses in the value-different and in the value-to-control compared with the value-same condition, *F*s(1, 177) > 12.83, *p*s <.001, *η*^2^_p_s >.06, but no significant difference between the value-different and the value-to-control conditions, *F*(2, 177) =.32, *p* =.569, *η*^2^_p_ <.01. Additionally, a main effect of Experiment was observed, *F*(1, 177) = 13.30, *p* <.001, *η*^2^_p_ =.07, indicating slower overall RTs in Experiment 1 (M = 868.71, SD = 151.34) compared with Experiment 2 (M = 816.08, SD = 119.86). As anticipated, no interaction between Distractor Change and Experiment was found, *F*(2, 354) =.39, *p* =.665, *η*^2^_p_ <.01.

**Fig. 5:**
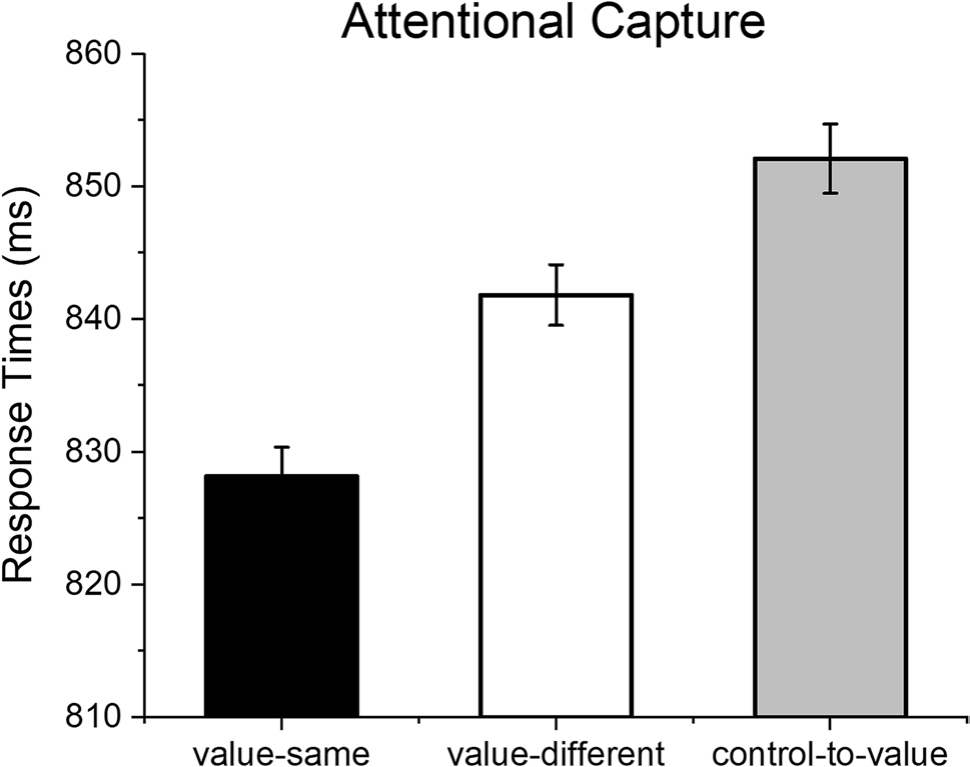
The effects of attentional capture (from a preceding trial in different conditions to a valued distractor). The distractor value (high-value, low-value, unrewarded) in the N trial in this analysis is the same across the three conditions (value-same, value-different, control-to-value)

In the second analysis (attentional capture; Fig. 5), the aim was to investigate the extent to which valued distractors captured attention in the actual trial, depending on the distractor that was presented in the previous trial. For this reason, distractor value (either high or low) was kept constant in the actual trial, while it was varied in the previous trial to be of the same value (value-same), of a different value (value-different), or a control stimulus (control-to-value). As in the previous analysis, the rationale was that if attentional capture is driven by the current distractor condition (and by intertrial priming), then one can expect fastest RTs (minimal attentional capture) for the value-same condition (intertrial priming), and similarly slower RTs for value-different and control-to-value conditions. On the other hand, if value contributes to the modulation of attentional capture, then one can expect a graded modulation of attentional capture, with fastest RTs for value-same condition, intermediate for value-different, and slowest for control-to-value ones. A main effect of Distractor Change was observed, *F*(2, 354) = 30.18, *p* <.001, *η*^2^_p_ =.14, indicating that RTs increased from the value-same to the value-different condition, *F*(1, 177) = 24.14, *p* <.001, *η*^2^_p_ =.12, and from the value-different to the control-to-value condition, *F*(1, 177) = 9.85, *p* =.002, *η*^2^_p_ =.05. Additionally, a main effect of Experiment was observed, *F*(1, 177) = 13.25, *p* <.001, *η*^2^_p_ =.07, indicating slower overall RTs in Experiment 1 (M = 873.10, SD = 151.10) compared with Experiment 2 (M = 820.65, SD = 119.67). As in the previous analysis, no interaction between Distractor Change and Experiment was found, *F*(2, 354) =.36, *p* =.690, *η*^2^_p_ <.01.

Finally, as value-same and value-different conditions were the same in the two analyses, we aimed to directly compare RTs in the control-to-value and in the value-to-control conditions. Responses were significantly slower in the control-to-value condition compared with the value-to-control condition, *F*(1, 177) = 12.22, *p* =.001, η^2^_p_ =.06.

### General discussion

Here, we examined the effects of learned value on the sequential modulation of attentional capture using a value-driven attentional capture paradigm (Mine & Saiki, 2015). In both experiments, highly significant distractor value repetition effects were observed, along with an interaction of current and previous distractor value, which was similar for monetary rewards (Experiment 1) and sustainability-related rewards (Experiment 2).

In both Experiment 1 and Experiment 2 we observed highly significant effects of change/repetition of distractor value from trial N-1 to trial N, with faster responses when distractor value was repeated compared with when it changed. This result is consistent with the literature that examined intertrial priming (e.g., Becker et al., 2009; Kristjánsson et al., 2007; Lamy et al., 2006; Leber & Egeth, 2006; Maljkovic & Nakayama, 1994), and in particular with those studies that observed that the repetition of distractor features or dimensions enhance performance (Eimer et al., 2010; Feldmann-Wüstefeld & Schubö, 2016; Kristjánsson & Driver, 2008; Lamy et al., 2008; Maljkovic & Nakayama, 1994). Concerning the effects of value on distractor repetition, we asked whether repetition effects are more sensitive to distractor condition or to distractor value. In the feature-change analysis, we reasoned that if distractor value contributes to repetition effects, then different (graded according to distractor value change) effects should be observed when comparing value-different with value-to-control stimuli. Instead, we observed that while repetition of the same distractor value from trial N-1 to trial N did speed up responses, slower and similar RTs were observed for different value and for unrewarded distractors. This result is particularly interesting as it suggests that distractor features rather than distractor value might be coded into an episodic representation, eventually speeding up responses when distractor features are repeated in successive trials.

Here, we investigated the extent to which distractors captured attention in the actual trial, resulting in performance slowing. Attentional capture interacted with previous distractor value in two ways: first, attentional capture by valued distractors compared with unrewarded ones in the actual trial was only observed when the previous trial was an unrewarded trial. Second, even when controlling for the effects of repetition of unrewarded trials (attentional capture analysis), responses to reward-related distractors were slower when they were preceded by unrewarded distractors, compared with when they were preceded by distractors of a different value. Altogether, these results suggest that attentional capture is sensitive to the retrieval of value of the previous trial, in that having had a rewarded distractor in the previous trial (even of a different value) diminishes attentional capture compared with unrewarded distractors. The present results are consistent with a cognitive control account (e.g., Frings et al., 2020; Hommel, 2004), in which feature repetition (in this case, distractor value) leads to a reinstatement of a previous control state that limits irrelevant or incongruent information from interfering with the activity that is being carried out. Models of binding-retrieval mechanisms suggest that features of both target and distractor stimuli can be integrated into an episodic memory that can result in attentional control when retrieved (Frings, 2011; Frings & Moller, 2010; Frings & Rothermund, 2011; Frings et al., 2007; see also Giesen et al., 2012; Hommel, 1998, 2005, 2007; Moeller et al., 2012). In a previous study, Hickey and colleagues (2010) used a single-phase approach based on the additional singleton paradigm (Theeuwes, 1991) in which the magnitude of reward feedback was manipulated at the end of each trial. Results showed that high-reward trials were followed by a reduced attentional capture by a singleton distractor if the color scheme of targets and distractors was maintained as similar, but the opposite result was observed if the color scheme changed (Hickey et al., 2010, 2014). Our results are in line with these findings in that they suggest that attentional mechanisms can be adaptively adjusted to optimize visual perception as a function of recent reward history, since we observed that the repetition of any valued condition between the ongoing and the preceding trial reduced attentional capture.

Taking together the results of the feature analysis and of the attentional capture analysis, here the repetition/change of value (valued/not valued) from one trial to the next did not affect overall RTs (feature-change analysis), but modulated the effects of attentional capture in the actual trial (attentional capture analysis). For distractors, the perceptual processing of salient but task-irrelevant features has been suggested to be less likely to capture attention if these features are repeated; it may be easier to ignore a distractor feature that is just be ignored. This facilitatory effect of distractor repetition has been explained in terms of attentional suppression (Barras & Kerzel, 2017; Gaspelin & Luck, 2018; Gaspelin et al., 2015), a mechanism that helps resolve the competition for attentional resources between items presented simultaneously in visual search tasks (Feldmann-Wüstefeld et al., 2021). Interestingly, attentional suppression can be modulated in sequential contexts (Feldmann-Wüstefeld & Schubö, 2016; Feldmann-Wüstefeld et al., 2016), and the present results expand previous results on attentional suppression, by observing that the repetition of reward-associated distractors, regardless of the exact repetition of value, can dampen distractor capture in the actual trial. Moreover, studies employing electrocortical measures of attentional capture and suppression observed that reward modulated the Pd (distractor positivity, Hickey et al., 2009), a component of the event-related potentials (ERPs) associated with attentional suppression (Sawaki et al., 2015; Taylor & Feldmann-Wüstefeld, 2024). Future studies may employ ERPs to clarify the role of attentional capture and suppression in the interaction between repetition and reward.

Here, in both experiments we observed that while the low-reward repetition effects were only observed when two distractors of the same value were repeated (low-reward distractor followed by low-reward distractor), a generalization effect was also observed for high-reward distractors. More specifically, faster responses were observed when a preceding distractor reward of either magnitude (low or high) was followed by a high-reward distractor. This result suggests a response facilitation which is prompted by the matching of a current high-reward stimulus and a preceding distractor of either high or low reward. Although unexpected, this result seems in line with evidence showing that negative stimuli predicting loss (here, distractors that threaten to prevent one from earning a high reward, i.e., negative punishment) can induce wider generalization gradients and reduce perceptual discrimination ability (Resnik et al., 2011; Shalev et al., 2018; see also Bruns et al., 2014). However, further studies are needed to explore this possibility.

In the present study, evidence for value-driven attentional capture was observed and replicated in both experiments when the present trial was preceded by an unrewarded condition, with attentional capture by rewarded (both high and low reward) compared with unrewarded control distractors (e.g., Anderson, 2016; Anderson et al., 2011). As both rewarded and unrewarded distractor colors were presented during the learning phase, the observed effects can be attributed to reward history and not to selection history (Anderson & Halpern, 2017; Le Pelley et al., 2015; Marchner & Preuschhof, 2018; Mine & Saiki, 2015). Notably, several studies observed VDAC regardless of previous trial condition manipulation or control (e.g., meta-analysis by Rusz et al., 2020); however, previous studies showed that the distracting value of rewards depends on the context in which they are perceived (Saez et al., 2017; Seymour & McClure, 2008). Future research could explore the extent to which learned value impacts sequential effects by employing a single-phase procedure that isolates and exclusively measures value-dependent effects (e.g., Le Pelley et al., 2015). Similarly, the present paradigm may be extended either by introducing a fourth condition in the test phase, featuring a completely novel distractor color not seen in the training phase that may be a control for selection history, or by using two distinct colors associated with high, low, and unrewarded rewards, therefore dissociating reward repetition from color repetition.

The results of both experiments are in line with past literature on reward and attentional capture, suggesting that stimulus feature-reward associations learned in one task transferred to another task, affecting the deployment of visual attention (Anderson et al., 2011; Anderson & Yantis, 2013; Anderson, 2016; Anderson & Halpern, 2017; Jiao et al., 2015; Le Pelley et al., 2015; Mine & Saiki, 2015). Specifically, regardless of the type of reward (monetary vs. environmental sustainability) used here, the present deployment of visual attention in visual search was modulated by the value defining distractors in the preceding trial. It is important to highlight that in both experiments, the reward was purely symbolic: In Experiment 1, most participants received feedback indicating a virtual gain (only about 18% of participants received a tangible monetary reward based on their task performance), and in Experiment 2, participants received feedback indicating that they were supporting an association fighting pollution. The crucial difference between the two experiments in this context is that monetary rewards are likely to be perceived as valuable by almost everyone, whereas the perception of value related to environmental sustainability may vary significantly between individuals. Nevertheless, the effects of environmental sustainability rewards mirrored the effects of monetary reward on search performance, suggesting that the principle of value-driven attention can be extended to other forms of incentives that have a symbolic meaning whose value is not recognized by everyone, but rather varies considerably between individuals. In terms of the differences between the two types of incentives, while monetary incentives modulated responses during learning in Experiment 1, the same effect was not observed in Experiment 2 in which sustainability-related incentives were given. In this respect, previous results in the literature describing value-driven attentional capture are mixed, with some studies showing performance facilitation by reward during learning (Anderson & Yantis, 2012; Failing & Theeuwes, 2014; Kiss et al., 2009; Krebs et al., 2011; Marchner & Preuschhof, 2018; Sha & Jiang, 2016), and other studies failing to observe the same effect (e.g., Anderson, 2016; Anderson et al., 2011, 2013, 2014, 2016; Miranda & Palmer, 2014; Roper et al., 2014).

### Conclusions

Here, we examined the role of learned reward in sequential modulation of attentional capture. Value-signaling distractors, if re-encountered, reduced attentional capture in the current trial, and this happened even for rewarded distractors of different values (e.g., high-value followed by low-value, and vice versa). These results are consistent with the view that items imbued with value represent a particular class of relevant information that modulates the interaction between memory processes and attentional capture. Moreover, sequential modulation of attentional capture by previously rewarded distractors was similar for different forms of incentives, suggesting that the role played by symbolic and domain-aspecific forms of reward representation is more important compared with that played by individual-specific reward representations.

## Data Availability

Subject-level aggregate data for both experiments are available via the Open Science Framework at: https://osf.io/c4yhb/?view_only=7134815235054532a3d6ca53f66aa9f7
